# Home Garden With Eco-Healing Functions Benefiting Mental Health and Biodiversity During and After the COVID-19 Pandemic: A Scoping Review

**DOI:** 10.3389/fpubh.2021.740187

**Published:** 2021-11-11

**Authors:** Xindi Zhang, Yixin Zhang, Jun Zhai

**Affiliations:** ^1^Department of Landscape Architecture, Soochow University, Suzhou, China; ^2^Collaborative Innovation Center of Architecture and Urban Environment of Soochow University-Suzhou Yuanke; China-Portugal Belt and Road Cooperation Laboratory of Cultural Heritage Conservation Science, Research Center of Landscape Protection and Ecological Restoration, Soochow University, Suzhou, China

**Keywords:** green environments, biodiversity, ecosystem services, nature-based-solution, plants and animals

## Abstract

The COVID-19 pandemic has led to tremendous impacts on human lives and society, which are not only because of negative effects on people's mental health due to isolation policies and physical distance for mitigating the spread of SARS-CoV-2, but also because the incident post-acute sequelae of the coronavirus will cause mental disorders. A green environment is a health resource, which cannot only benefit human physical and mental health, but also increases biodiversity, contributes to flood mitigation, and cools urban areas. A home garden, as a kind of small green space, can provide ecosystem services with eco-healing functions in reducing mental stress during the isolation period of the COVID-19 pandemic through the garden itself and physical activities in it. Such an eco-healing approach within a mini-therapeutic landscape can also benefit biodiversity by enhancing plant diversity in residence and increasing biodiversity at a large scale. In this article, we propose a conceptual framework describing a home garden as “ecological medicine” with healing functions to improve mental health, as well as indirectly enhancing urban biodiversity. A home garden, as a mini-type of green landscape with biodiversity content, allows people to get close with nature so that it can promote comfortable and natural feelings during the pandemic. Furthermore, such an eco-healing home garden approach benefiting urban biodiversity can meet the challenges in maintaining environmental and mental health in post COVID-19 pandemic recovery, as well as preparing unknown next-surge risks with potential isolation regulations.

## Introduction

The 2019 coronavirus disease (COVID-19) pandemic sweeping the world has had a profound impact on all aspects of human society. It was calculated that the global cumulative number of people suffering from COVID-19 was over 157 million, and the death rate had reached 3.29 million in May 2021 (1). Throughout the COVID-19 pandemic, countries have enforced stay-at-home advisories or shelter-in-place and border control policies to control the spread of the coronavirus SARS-CoV-2. And now, as the pandemic is almost under control, policies have changed, stating that residents or visitors entering from abroad should be tested for COVID-19 and quarantined for more than 14 days immediately.

The policies during the pandemic reduced the likelihood of people getting along with others (2, 3), and the loneliness of isolation accompanied by the fear of the COVID-19 pandemic, the stress of economic downturn, and the increasing amount of unemployment also affected people's mental health (4–9). It indicated that the proportion of the population suffering from depression during the COVID-19 pandemic in the U.S. was three times higher than before (4, 10). And data collected from the Household Pulse Survey showed that more than 35% of adults in the U.S. experienced anxiety or depressive disorder during February to March 2021, while the data collected from January to June 2019 were 8.2% for anxiety disorder, 6.6% for depressive disorder, and 11.0% for anxiety or depressive disorder. A high-dimensional approach also identified that incident post-acute sequelae of the COVID-19 included neurocognitive disorders and mental disorders (11). In the long run, mental health risks associated with the COVID-19 pandemic can be more harmful than the virus itself (12, 13). Thus, it is necessary to find a green and sustainable solution to meet the challenges.

For mental disorders, the traditional treatment is psychiatric medication (14–16). However, the use of medications can have unwarranted side effects. In addition, traditional psychiatric treatment may be associated with stigmatizing attitudes in general, while complementary or alternative therapies are widely accepted (17), such as plant and horticultural therapy.

Plant-based or gardening-based horticultural therapy can lead to a reduction in the prevalence of distress such as depression, stress, and anxiety, and they can improve mental health (18, 19). The rehabilitative effects of horticultural therapy are based on the theories of green spaces' healing functions to benefit people not only physically but also psychologically (20–24). These healing functions depend on the health of the human environment, the functions of the ecosystem (25, 26), and biodiversity (27). The promoting effects are positively correlated with the increase of greenness and proximity to green space (28). And the main benefit is that more physical exercise in green space can have effects on reducing greenhouse gas emissions and enhancing more appreciation of natural environment and biodiversity (29). The established aspects of green space with biodiversity can make significant contributions to physical and mental health (25, 27).

Based on the theories of psychological treatment and horticultural therapy, the concept of “ecological medicine,” which is one type of nature-based solution, has emerged for improving mental health through natural space. “Ecological medicine” mainly refers to a home garden and home gardening in this article because the isolation policies reduced the opportunities of access to public green space during the COVID-19 pandemic, and a home garden can provide mental health services in limited areas. Especially, “ecological medicine” has not only therapeutic effects, but also ecological and sustainable effects, such as lowering the temperature, improving air quality, and increasing urban biodiversity. The objectives of this article dealing with the home garden are: (a) reviewing and examining how a home garden, a kind of green space, can contribute to mental health during the COVID-19 pandemic and in the future, and (b) exploring the positive effects of home gardening, a kind of physical activity, on mental health during the isolation period.

## Conceptual Model

**Figure 1:**
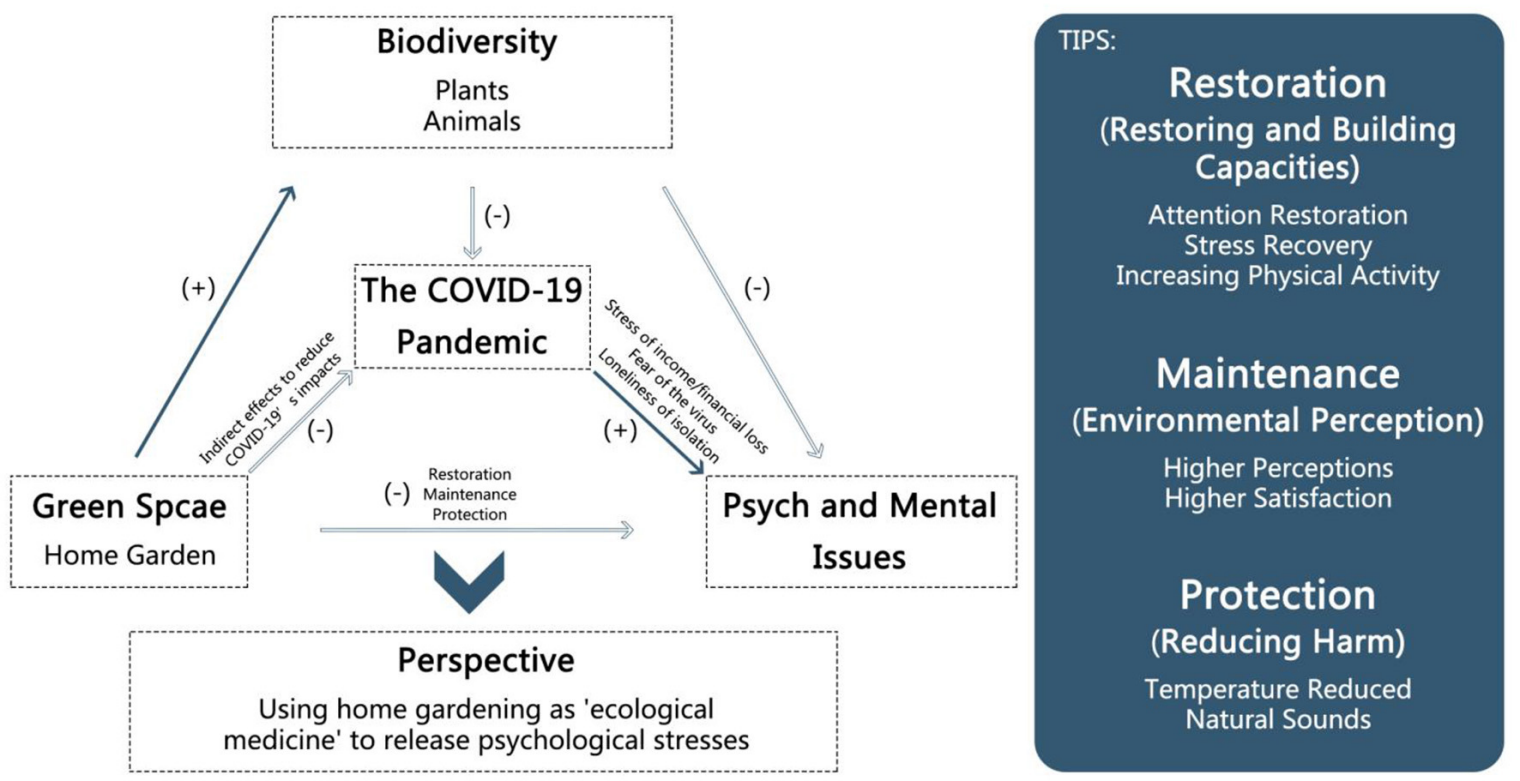
The conceptual model of effects of green space/home garden on reducing mental problems under the pandemic (scenario) and benefiting biodiversity. It shows the logical framework of the green space with eco-healing effects. To control the COVID-19 pandemic, stay-at-home advisories or shelter-in-place policies were published. But, on the other hand, some mental issues including the feelings of stress, fear, and loneliness increased. Green space as one nature-based solution has been proven to relieve mental problems and stress through restoration (restoring and building capacities), maintenance (environmental perception), and protection (protection). And it also increases urban biodiversity through plants and animals. Due to the low likelihood of people accessing public green spaces, a home garden is mentioned. So the home garden, a kind of green space at a small scale, is good for mental health during the COVID-19 pandemic and has an indirect positive effect on combating the challenges of the pandemic. Thus, the idea of using green space, a kind of natural solution, to improve mental health and urban biodiversity not only during the pandemic but also in daily life is raised.

For achieving mental health and benefiting biodiversity through green space, a conceptual model of green space (home garden) for biodiversity and mental health under the COVID-19 pandemic is proposed (Figure 1). The logical framework of green space (home garden) to improve mental health is linked to its eco-healing effects as “ecological medicine” in reducing mental problems and stress which are caused by being unable to visit green spaces and parks.

## Methods

### Strategy for Selection of Articles

In order to carry out the scoping review, electronic journal databases (Web of Science, Science Direct, Google Scholar, Johns Hopkins University and Medicine, WHO, and other databases) were used to collect literature and other information. Firstly, over 200 articles in the last 20 years were selected by searching topic words including human health (set 1: “the COVID-19,” “psychological health,” “mental health,” or “morbidity”), green space (set 2: “green space,” “home garden,” “balcony garden,” or “biodiversity”), and activities (set 3: “home gardening”). At the same time, psychological health or mental health was combined with some keywords like COVID-19, green space, home garden, balcony garden, and home gardening. For a thorough understanding, references in relevant articles were also reviewed. Secondly, to be included in the article, literature had to meet the following criteria through a review of the abstract, introduction, and methods: (a) first-hand research articles were preferred, (b) findings were directly applicable to the mental health promotion of home garden or home gardening, and (c) articles had high impact. Based on these, over 100 articles from 68 scientific journals in health, medicine, ecology, landscape, environmental sciences, and other relevant areas were finally selected. Lastly, we divided the collected articles into two categories: (a) the positive influence of a home garden on mental health, and (b) the promotion of home gardening on mental health (Table 1).

**Table 1:** The table shows some literature discussing aspects of **(A)** the positive influence of a home garden on mental health; **(B)** the promoting effects of home gardening (a kind of physical activity) on mental health.

**Study characteristics**	
**(A) The positive influence of a home garden on mental health**	
**keywords:** green space, home garden, balcony garden, biodiversity, psychological health, mental health	
**Aspect**	**Study**
(i) More areas with green space can result in better mental health; (ii) More frequently entering into green space can lead to fewer mental problems; (iii) Green spaces improve mental health through three main stages, including restoration, maintenance, and protection; (iv) Biodiversity makes significant contributions to mental health; (v) Contact with nature can reduce stress, anxiety, and other negative emotions.	Almanza et al. (30); Annerstedt et al. (31); Arslan et al. (32); Campbell et al. (25); Carrus et al. (33); Cox et al. (34); Cracknell et al. (35); Dadvand et al. (28); De Jong et al. (36); De Vries et al. (37); DeSchriver and Riddick (38); Dong et al. (39); Dzhambov et al. (40); Engemann et al. (20); Fuller et al. (41); Fuller and Gaston (42); Gascon et al. (43); Gascon et al. (44); Goldstein et al. (45); Grahn et al. (46); Han, (47); Hartig et al. (21); Hartig and Kahn (48); Jackson (26); Kumar et al. (22); Li et al. (49); Markevych et al. (50); Mitchell (51); Mytton et al. (52); Sarkar et al. (53); Taylor and Hochuli (27); Tzoulas et al. (23); Ulrich et al. (54); Van den Berg et al. (24); Van Renterghem (55); Wells (56); White et al. (57); Wild-Eck (58); Wilkie et al. (59); Wilkie et al. (60); Wu et al. (61); Young et al. (62).
**(B) The promotion of home gardening on mental health**	
**keywords:** home gardening, psychological health, mental health	
**Aspect**	**Study**
(i) Home gardening can be good for mental health through relaxation and restoration; (ii) Home gardening can strengthen human contact with the natural environment; (iii) Home gardening can improve physical health to promote mental health.	Chalmin-Pui et al. (63); Chang et al. (64); Corley et al. (65); Dennis et al. (66); Dzhambov et al. (67); Korn et al. (68); Lachowycz and Jones (69); Lakhani et al. (70); Pouso et al. (71); Sofo and Sofo (72); Soga et al. (73); Theodorou et al. (74)

### The Procedures

As it was difficult to conduct a systematic review or meta-analysis, we decided to conduct a scoping review. Based on the literature we collected and selected, we summarized two broad themes to organize the review (Table 1). And this study mainly focuses on examining if a home garden and home gardening promote mental health directly or indirectly during and after the COVID-19 pandemic:

(1) To think about the solutions of mental health needs during the lockdowns period of the COVID-19 pandemic, the articles discussing the benefits of green space (home garden) or gardening on mental health were searched and collected. The mental health benefits of exposure to nature can be found in both direct and indirect aspects. The direct aspect is about the benefits of nature factors, such as natural colors, natural sounds, and animals, while the indirect aspect is about benefits like increasing physical activities.
(2) The mechanisms of a home garden and home gardening on mental health promotion were also analyzed by summarizing and exploring the benefits of gardens' components including water, plants, animals, and so on. And then we developed a framework about the benefits between home garden/home gardening and mental health. The framework can be used not only during the COVID-19 pandemic but also after it.

## Results

### Overview: Effects of a Home Garden and Home Gardening on Promoting Mental Health

Green spaces including home gardens and balcony gardens have positive influences on mental health (Table 1A), and home gardening also has a promoting effect on mental health (Table 1B). Thus, during and after the COVID-19 pandemic, green space (home garden) will be “ecological medicine” for mental health.

### Analysis: Odds Ratios for Describing the Positive Effects

**Figure 2:**
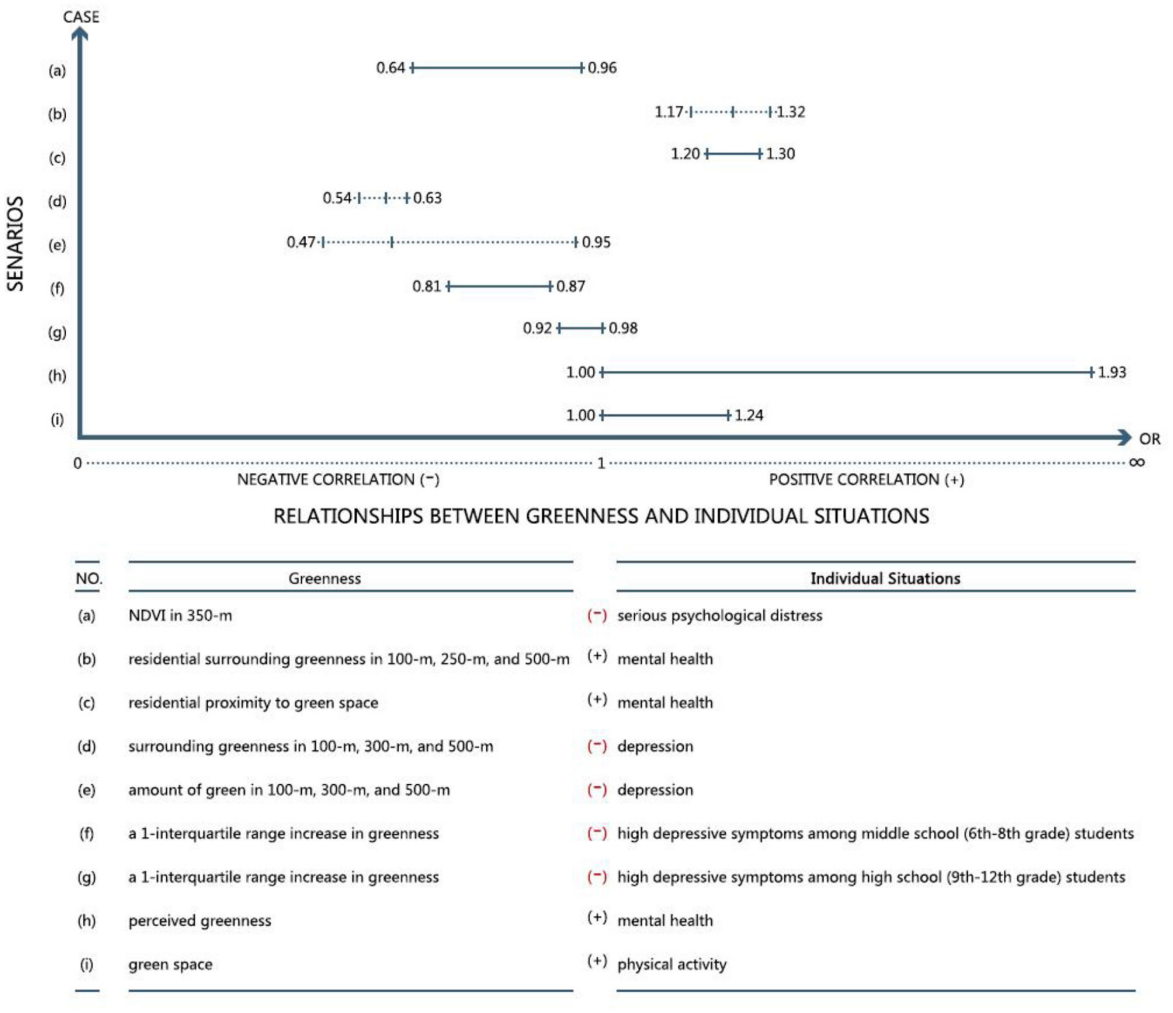
By searching data from the previous studies, the relationship between greenness and individual situation (see Appendix A for references) is reflected by odds ratios (ORs). If the OR < 1, it means that there is a negative correlation between the two factors. And it will be positive between two factors if the OR is more than one. There are four pairs of relationships that are positively correlated: (b) residential surrounding greenness and mental health, (c) residential surrounding greenness and mental health, (h) perceived greenness and mental health, and (i) green space and physical activity, which indicate that greenness will promote mental health and physical activity. In addition, negative correlations of scenarios with mental problems are: (a) NDVI over 350 m, which represents the greenness assessed by the Normalized Difference Vegetation Index (NDVI) surrounding a residence over 350 m, (d) surrounding greenness over 100, 300, and 500 m, (e) amount of green over 100, 300, and 500 m, and (f) (g) a 1-interquartile range increase in greenness. The solid lines represent the range of the relationship, and the dashed lines indicate the potential range of the relationship.

Green spaces have positive effects on improving mental health (42, 53). People living closer to urban green spaces are often associated with lower mental distress (57) because of the buffering effect of green space in reducing negative feelings, such as depression and anxiety (Figure 2). And it can promote mental health through sight, hearing, touch, and smell. The relationship between greenness and the individual situation of Figure 2 is reflected by odds ratio (OR) which uses Equation (1):


(1) $$\begin{array}{r} OR=\frac{A/B}{C/D}=\frac{AD}{BC} \end{array}$$

A: number of exposed persons in the case group.
B: number of non-exposed persons in the case group.
C: number of exposed people in the control group.
D: number of non-exposed people in the control group.

A home garden acts as an important kind of green space in a balcony or courtyard, it can also provide space for home gardening so that people can reduce mental problems through physical exercise. Green space complexity can vary dramatically in contributing to benefiting human mental health, and green spaces with higher species richness can also have greater mental benefits (41).

## Discussion

### The Home Garden as “Ecological Medicine” During the COVID-19 Pandemic

In order to control the spread of the COVID-19 pandemic, governments quickly took protective measures, such as closing public places like shopping malls, gyms, and schools, and also discouraging social gatherings. In addition, many countries took stricter measures, such as imposing total lockdowns or even curfews (75). However, such measures have led to a more sedentary shift to unhealthy lifestyles through people staying away from work, schools, or gyms (76, 77).

A study investigating the effects of stay-at-home policies taken during the COVID-19 pandemic on human eating behaviors, physical activities, and body weight showed that participants spent less time exercising and more time sitting or lying each day, which would lead to weight gain (78). Therefore, policy interventions to ensure control of the COVID-19 pandemic should be accompanied by attention to maintaining a healthy lifestyle.

In this context, a home garden, an important part of green space, becomes the preferred option to deal with the dual pressure of lockdown policies and mental health needs. A study of physical and mental health effects of a home garden using among older adults in Scotland during the COVID-19 pandemic suggested that a home garden had positive benefits on subjective well-being, including physical and mental health (65).

It has also indicated that a home garden promotes mental health through not only itself but also activities such as home gardening. On the one hand, a home garden, along with other types of green space, provides opportunities for people to interact directly with nature which is beneficial to mental health (79, 80). A home garden can provide people with sunshine and fresh air, which can have indirect mental health benefits, including helping with sleep and eating patterns (81, 82). In addition, spending time in a home garden involves physical activities, which can promote physical strength, health, and flexibility, and additionally, provide mental health benefits (83). And it also shows that spending time in a home garden can provide opportunities for people to interact with their neighbors while obeying social distancing, thereby promoting a sense of community and social connection, and also indirectly improving mental health (37) especially during the lockdowns.

Thus, a home garden as a potential health resource can play the role of “ecological medicine” during the COVID-19 pandemic. Following on, this article will focus on discussing (a) the mechanisms of a home garden on mental health, and (b) the positive effects of home gardening on mental health.

### The Mechanisms of a Home Garden Promoting Mental Health During the COVID-19 Pandemic

A home garden is a small ecosystem which can provide ecosystem services, and enhance human health at a small scale, including improving mental health, mitigating allergies, and reducing all-cause, respiratory, cardiovascular, and cancer mortality (84). And it also shows that a home garden can benefit mental health directly through three main stages: (a) restoration, i.e., recovery from stress (37), (b) maintenance (40, 44, 50), and (c) protection (37, 40, 44, 50).

People with mental problems will show high stress, low attention, and low interest. A home garden has been proved to improve mental health through restoring capacities, which means attention restoration (54, 59, 60), stress recovery (46, 85), and increasing physical activity (51, 52). A study taken among 116 college students in Taiwan showed that 15-min exercise in an area with at least 40% visible greenery was good for attention restoration (47). Higher green levels will result in lower stress levels (85), and the more time and higher frequency of access to green space can also lead to less stress (46). In addition, greenness is also positively correlated with the frequency of physical activity (52). A survey taken in California showed that children would exercise more frequently in locations with more green space than in locations with less green space (30).

And maintenance means that higher perceived green space and satisfaction of the residential environment can potentially maintain mental health (36, 39, 69), and the higher levels of greenness will result in higher levels of satisfaction. The greenery visible from home and in the neighborhood was associated with a decrease in depression and anxiety symptoms. More houseplants indoors or in gardens are associated with better mental health, which supports the hypothesis of the mental health-support effects of indoor greenery (67).

During the COVID-19 pandemic, in addition to the pandemic itself, some environmental factors, such as a climate change-related urban heat island, can also affect mental health. Research has indicated that climate change can affect mental health indirectly through affecting physical health, for example, extreme heat can cause heat stroke in vulnerable people and lead to mental health problems, including an increased suicide rate (86). Green spaces with trees and vegetation can lower land surface temperature by providing shade and through evapotranspiration, and a study in Suzhou, China showed that a 10% increase in green space coverage was associated with a 1.41°C reduction in surface temperature (61), which could reduce the urban heat island effect to release the pressure of heat stroke. In a similar way, a home garden and active interactions with indoor plants can also be an interactive ecological buffer that reduces mental stress through suppressing sympathetic nervous system activity and promoting feelings of comfort, soothing, and naturalness (87), which then, improves mental health during the pandemic.

In general, a home garden can provide the sustainable use of natural resources and ecosystem services through plants, in terms of health, economic, productive effects (72), and it can also make a beneficial contribution to urban biodiversity conservation (88). Though the lockdowns during the pandemic reduced the likelihood of people encountering outdoor green space, the home garden can be a new way to replace public green space and improve mental health through restoration, maintenance, and protection.

### The Positive Effects of Home Gardening, a Kind of Physical Activity, on Mental Health

Research has found that mental health is positively associated with the frequency of access to green space and the presence of green window views (the greenery outside the window brought by the community greenery, balcony greenery, courtyard greenery, vertical greenery, and so on) (73), which can also benefit recovery from sickness (89). Based on this, a home garden can be a source of greenery with a significant mitigating effect on mental problems through these two aspects (66), especially during the isolation period of the COVID-19 pandemic.

An online survey taken in April 2020 collected 1,491 adults' reports and showed that there were negative changes in physical activity during the COVID-19 pandemic which was associated with higher depression, anxiety, and stress symptoms (90). In view of this phenomenon, a home garden can be a choice for increasing physical activity, such as home gardening, which is good for mental health (45, 70). And a survey among 5,766 gardeners and 249 non-gardeners within the UK showed that gardening at least 2–3 times a week could result in better mental health (63). There was also a similar survey taken during the Italian lockdown from March to May 2020 indicating that participation in gardening activities could promote mental health by reducing stress from the COVID-19 pandemic (74). Due to the isolation policies during the COVID-19 pandemic, people could not go to the park to relax, so the home garden became a good alternative for people to undertake physical activities within a limited area.

While doing home gardening, people can improve mental health not only through taking part in physical activities, but also through getting in touch with nature. Evidence has also shown that plant fragrance or color can improve mental health (32, 41, 58, 91), and natural sounds can relax people and support recovery by suggesting proximity to nature (31, 55). In addition, some components and elements of a home garden, such as water (92) and flowering plants (49), were proved to be better for mental health. Besides, medicinal and aromatic plants are also good choices for home gardens which can stimulate the senses of garden users (32).

The isolation policies during the COVID-19 pandemic reduced communication among people and communities, as well as reduced people's access to public green space for exercise. A home garden, as a kind of green space which can be built in balconies, yards, or other areas, can increase the likelihood of exposure to green space and increase physical activity so that it can benefit mental health not only in high-income areas but also in low-income communities (68).

Therefore, a green space/home garden can play a role of “ecological medicine” and a natural solution to reduce mental problems during and after the pandemic. And home gardening has also been proved to be good for mental health (93). In the lack of access to green space (69) during the lockdown period of the COVID-19 pandemic, a home garden, a small-scale green space, becomes a rational alternative for contacting greenery (65) by increasing the frequency of access to green space and increasing physical activities.

## Recommendations

### “Ecological Medicine” for Achieving Mental Health Through Natural Solutions Using a Home Garden

**Figure 3:**
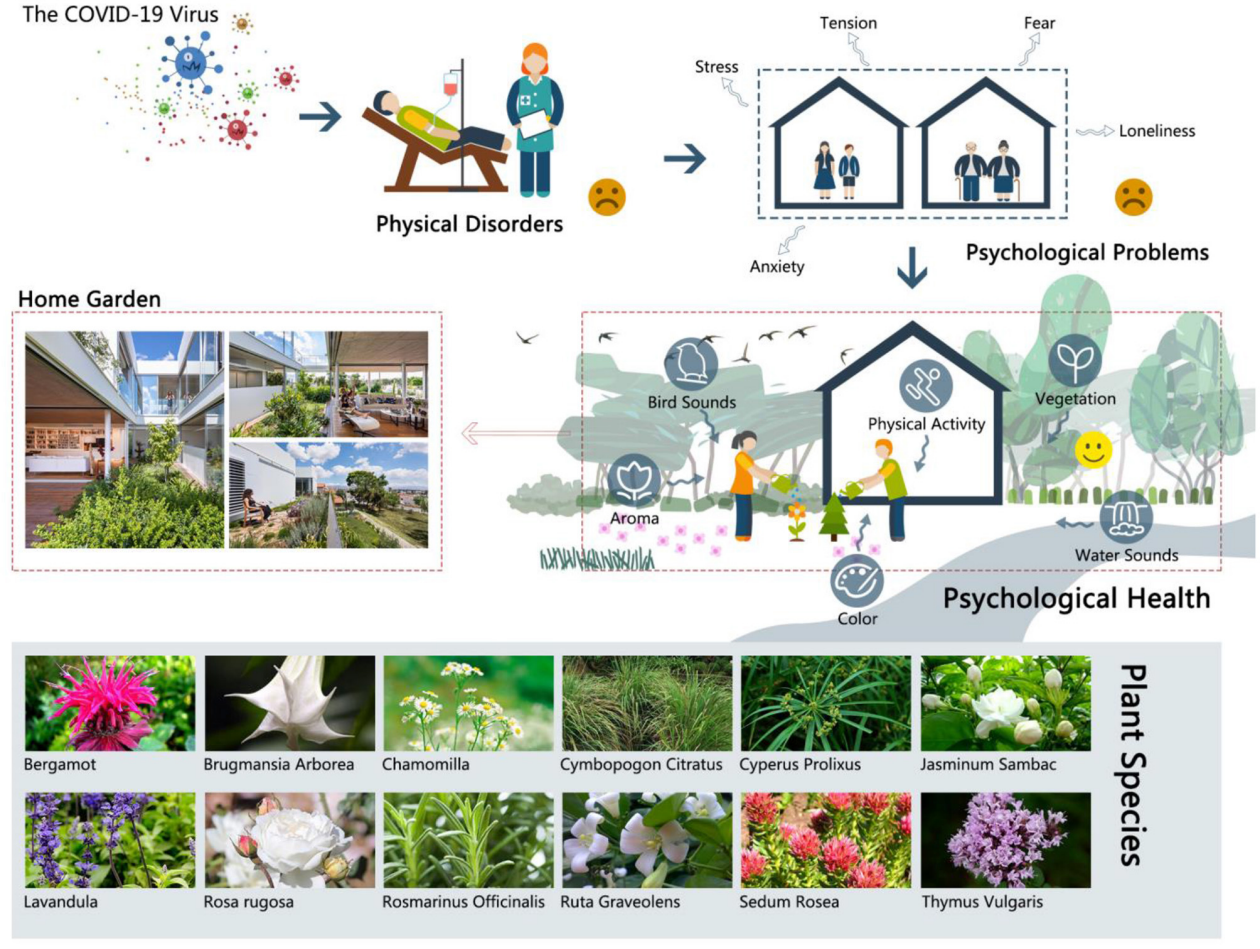
The diagram shows the mechanisms of “ecological medicine” in which the logical framework is discussed in Figure 1. And the diagram also shows some plant species in a home garden for mental health. The government gives stay-at-home advisories or shelter-in-place policies in order to control the spread of the COVID-19 pandemic. However, people will feel stress, tension, fear, anxiety, and loneliness due to isolation from society. A home garden, a kind of green space, can improve mental health through natural sounds (birds sounds or water sounds), aroma, color, and vegetation, and it can also benefit biodiversity through more kinds of plants and animals. What is more, it can also increase the frequency of physical activity, such as home gardening, which can give people opportunities to get in contact with nature and release mental stress to improve mental health, as well as benefit biodiversity conservation. The benefits of these can be summarized into three stages: restoration (attention restoration, stress recovery, and increasing physical activity), maintenance (higher quality of green space and residential environment can potentially maintain mental health), and protection (home garden as a buffer between human and harm from the environment). And the pathway of this is called “ecological medicine” which is a kind of nature-based solution. Image (left-down picture of a home garden): https://mooool.com/the-garden-house-in-the-city-by-christos-pavlou-architecture.html.

The World Health Organization (WHO) defined human health as “a state of complete physical, mental, and social well-being and not merely the absence of disease or infirmity” in 1948, and the definition has not been amended since then. While fighting with the COVID-19 pandemic, we cannot ignore the increasing number and harm of mental problems. So “ecological medicine,” a kind of natural solution, will become a good choice to achieve mental health when a lot of medical resources are invested in the treatment of the virus (Figure 3).

“Ecological medicine” as a nature-based method can deal with issues of mental problems during the isolation and physical distance period through ecosystem services from a home garden. Some ecosystem services are related to human health and well-being, such as mitigating allergies, heat, noise, and air pollution, as well as improving mental health (84). A national survey taken in Singapore by online questionnaires among 1,262 people showed that high life satisfaction, which improved mental health, was positively associated with nature experiences and views from windows (64).

Many studies have indicated that a home garden can improve mental health through increasing physical activity (45, 63, 70). Flowering plants in a home garden have better effects on mental health than foliage plants. A study among 150 college students showed a 10-min image of an impact to participants in order to elicit stress. After 5 min of recovery, the participants were randomly divided into three groups: those that looked at (a) red geraniums, (b) only green leaves, and (c) nothing. The results showed that the participants significantly recovered from the pressure after seeing the red-flowered geranium, and it was very obvious compared with the other two groups (49).

Additionally, higher biodiversity was shown to be greater for mental benefits by a study taken in the UK among 79 participants (*N*_1_ = 39; *N*_2_ = 40) (35). And a home garden constitutes a key center for biodiversity conservation (94). In a home garden, medicinal and aromatic plants can significantly increase biodiversity (95) and be good choices for healing because they stimulate the senses of garden users to relieve mental disorders (32). According to the WHO (World Health Organization), (96), around 80% of people in all developing countries use medicinal plants as a primary source of health care and sometimes as the only available treatment. And some studies have shown that many kinds of plants, such as *Brugmansia arborea, Cyperus prolixus*, and *Ruta graveolens*, can be used to treat bad moods (95).

However, allergenic plants should be avoided in a home garden as they can be associated with the prevalence of allergy diseases (97), which will cause more serious mental distress. It has been indicated that exposure to pollen from some allergenic plants is associated with severe allergy symptoms (98). Therefore, we need to pay special attention to control the application of allergenic plants (especially flowers that undergo pollination by wind, e.g., plant species belonging to genera *Acer, Tilia, Betula, Populus, Platanus, Celtis, Aesculus, Thuja, Ulmus, Robinia*, and *Quercus*) (99) and increase the area of non-allergenic plants, so as to reduce the pollen concentration of allergenic plants in the air.

What is more, being surrounded by natural sounds (birds sounds or water sounds) has been proved to be helpful to reduce stress (31) through relaxing people and supporting recovery by suggesting proximity to nature (55). It has also been proved that animal watching not only makes participants lower their pulse rate and muscle tone, and increase their skin temperature, but also provides positive natural feelings to benefit mental health (38, 56).

In order to control the spread of the virus, the U.S. government established stay-at-home advisories or shelter-in-place policies to implement extraordinary physical distance interventions so that people could not access community green spaces, parks, and other public places for activities (8, 100). However, a home garden as a green space at a small scale has been proved to be beneficial to mental health by increasing the frequency of access to green space, views from windows, and physical activity. During the isolation period of the COVID-19 pandemic, a home garden became a new choice for people to get in touch with nature. So people could use their balconies or private courtyards for gardening and build their own home gardens to make contact with nature and release stress, and relieve loneliness and other mental problems. And in a home garden, flowering, medicinal, and aromatic plants will be better for mental health (32, 49). Natural sounds from birds or water are also proved to be good for mental health (31, 55). With these findings, people can design and build their home gardens to relax and exercise for their mental health.

During the COVID-19 pandemic, people could use this “ecological medicine” as a new way to maintain mental health through a home garden and home gardening which are less harmful and more sustainable ways to reduce stress, tension, fear, anxiety, and loneliness from the virus or the lockdowns and improve their mental health.

## Conclusions

The COVID-19 pandemic has disrupted the normal rhythm of life in the world over the past 17 months, and will continue to affect people's health for an uncertain period of time, especially mental health (101–107). Due to the shortage of mental health professionals and the sharply increasing demand for mental assistance or interventions for the general population (108–110), it is urgent and crucial to define rational nature-based practices that are developed for mental health care (111). The stay-at-home and lockdown policies during the COVID-19 pandemic have made home gardens a rational alternative choice for promoting mental health.

### Home Garden, a Small-Scale Green Space, Can Promote Mental Health With Home Gardening

Home garden/home gardening can be “ecological medicine” to provide mental health benefits through restoration, maintenance, and protection during the isolation policies. In home gardens, people cannot only get in close contact with nature through sensual messages (touching, seeing, smelling, and listening), but also divert their negative emotions through gardening and release the pressure of mental disorders. Additionally, home gardening along with urban green nature has great potential to be a “nature-based solution” for improving public mental health during the pandemic, and it can also indirectly contribute to the control of the COVID-19 pandemic.

### Biodiversity Increasing due to Home Gardens for Mental Health

Mental benefits are also positively associated with the biodiversity of plants and animals (33, 34, 62). There is also evidence that green spaces with wildlife may be better for mental health because they can provoke a sense of connection with the whole of nature (91), and afternoon bird abundances can benefit well-being through increasing interactions (34). A home garden can be a kind of small green space in urban areas benefiting mental health, and a complex network of home gardens in cities can provide a positive effect on improving urban biodiversity (112).

### “Ecological Medicine” Is an Efficient Healing Alternative to Improve Mental Health—Nature Experience as a Determinant of Mental Health

To deal with the negative effects of isolation and social distance policies on mental health, a home garden and gardening can be effective approaches for people re-connecting to green environments to provide a nature experience with eco-healing effects on maintaining mental health, and it may also reduce the health burden due to COVID-induced disability and sequelae across all age groups, including neurocognitive disorders and mental health disorders (11, 113). Natural sounds, vegetation color and shape, plant fragrance, and so on coming from a home garden can help improve mental health through sense organs by hearing, seeing, touching, and smelling (32, 41, 58, 91). A home garden, with flowers, animals (e.g., bees, butterflies), medicinal herbs, and so on, not only has the benefits of increasing green habitats and supporting urban biodiversity (plants, animals, and soil microbiome), but also has the advantages of being accessible at any time, with nature experience (gardening) effects on cognitive functioning, emotional well-being, and other multiple dimensions of mental health. Balconies, backyards, or courtyards can be designed and built with effective nature-based solutions with eco-healing effects to improve public mental health during lockdowns of the pandemic and indirectly help contain the COVID-19 pandemic. And in the future, such eco-healing home gardens can be an approach to meet challenges in mental health not only in post COVID-19 pandemic recovery, but also in risks of future unknown pandemic surges with isolation and physical distancing regulations.

## Author Contributions

XDZ and YXZ designed this study and wrote the manuscript. JZ discussed this manuscript. All authors contributed to the article and approved the submitted version.

## Funding

YXZ's research is partially supported by a grant (p113800618) of Soochow University-Suzhou Yuanke (SU-SY) Collaborative Innovation Center of Architecture and Urban Environment. This project was also supported by the National Key Research and Development Program of China (grant no. 2021YFE0200100; China-Portugal Belt and Road Cooperation Laboratory of Cultural Heritage Conservation Science).

## Conflict of Interest

The authors declare that the research was conducted in the absence of any commercial or financial relationships that could be construed as a potential conflict of interest.

## Publisher's Note

All claims expressed in this article are solely those of the authors and do not necessarily represent those of their affiliated organizations, or those of the publisher, the editors and the reviewers. Any product that may be evaluated in this article, or claim that may be made by its manufacturer, is not guaranteed or endorsed by the publisher.

## Appendix A

**Table A1:** **Odds ratios (ORs) for the association between greenness and individual situations.**.

**Model**			**OR**	**Author**
**NO**.	**Greenness**	**Individual situations**		
(a)	NDVI over 350 m	Serious mental distress	0.64–0.96	Wang et al. (114)
(b)	Residential surrounding greenness over 100, 250, and 500 m	Mental health	1.32, 1.25, and 1.17	Dadvand et al. (28)
(c)	Residential proximity to green space		1.20–1.30	
(d)	Surrounding greenness over 100, 300, and 500 m	Depression	0.54, 0.59, and 0.63	Gascon et al. (44)
(e)	Amount of green over 100, 300, and 500 m		0.95, 0.47, and 0.60	
(f)	A one-interquartile range increase in greenness	High depressive symptoms among middle school (6th−8th grade) students	0.81–0.87	Bezold et al. (115)
(g)		High depressive symptoms among high school (9th−12th grade) students	0.92–0.98	
(h)	Perceived greenness	Mental health	1.00–1.93	Sugiyama et al. (116)
(i)	Green space	Physical activity	1.00–1.24	Mytton et al. (52)

*Nine pairs of interrelationships were extracted from six articles, and whether the relationships between them were positive or negative was reflected by the odds ratios*.
